# Epidemiology of moderate and severe traumatic brain injury in Cairo University Hospital in 2010

**DOI:** 10.1186/1757-7241-21-S2-A4

**Published:** 2013-09-09

**Authors:** Tamer Montaser, Ahmed Hasan, Ahmed Ibrahim

**Affiliations:** 1 Cairo university hospital, Cairo, Egypt; 2 King Khaled University Hospital, Kingdom of Saudi Arabia; 3 Shobra General Hospital, Cairo, Egypt

## Background

Traumatic Brain Injury (TBI) is a contributing factor to approximately one third of all injury-related deaths in USA annually. Updated statistical records for TBI in Egypt are lacking. The current research is aiming for estimating the prevalence of TBI in Egypt in order to develop a comprehensive TBI prevention program.

## Methods

One year period (one calendar month every quarter of 2010) descriptive epidemiological study of moderate and severe TBI cases admitted to the emergency department, Cairo main university hospital. The Data collection sheet included personal data (age, sex and residency), incident related data (cause, nature and time of injury) and both; clinical and radiological findings.

## Results

Moderate and severe injuries account for 17.2% (844) of all TBI presented cases in the 4 months for the study. Male sex was predominantly affected 79% of cases. 63% of the cases were between 19 and 55 years old and the 2 main causes were fall from height (FFH) and motor vehicle collision (MVC) which account for about 64% of cases. 17% of cases were among pediatric group (1-18 years) and FFH was the leading cause with 34% followed by MVC and stuck by or against events with the same percentages (21%). Causes of moderate and severe TBI among seniors (above 60 years) were FFH (28%), MVC (24%), and Stuck by or against events (15%).

## Conclusion

Traumatic brain injury is a serious public health problem in Egypt. Further data interpretation over wider periods of time should be conducted for better understanding of TBI prevalence is highly recommended to develop effective injury prevention program. Inefficient recording should raise the concern to establish an optimal system for data recording and interpreting.

